# On Graves's Disease, with a Case

**Published:** 1887-03

**Authors:** J. Michell Clarke

**Affiliations:** Assistant Physician Bristol General Hospital; Demonstrator of Physiology at the Bristol Medical School


### ON GRAVES'S DISEASE, WITH A CASE.

Read before the Bristol Medico-Chirurgical Society

BY

J. MICHELL CLARKE, M.A., M.B. CANTAB.,

*Assistant Physician Bristol General Hospital; Demonstrator of Physiology at the Bristol Medical School.*

---

THE case that I am about to report is that of a girl, aged 18, who was born and always lived at Chepstow. The family history was good, the two preceding generations being long lived. She was one of a family of fifteen, and had four sisters and three brothers alive and well; seven brothers died in infancy—four of scarlet fever, one from hip disease.

She had always been perfectly healthy; had had no serious illness. Menstruated at 14; always regular. In 1885 she had headaches occasionally, with slight feeling of fulness at the throat, but was otherwise in her usual good health In June, 1886, she experienced during a menstrual period violent palpitation of the heart, severe frontal headache, pain in the præcordial region, with nausea and vomiting in the morning. The next period was regular, and without any abnormal symptoms; but since then she has seen nothing. She remained well until August 4th, when the palpitation returned—though not so severely as in June,—her eyes appeared to be growing larger, her throat commenced to swell and throb, her voice became more and more nasal in character, there was slight cough, and she rapidly lost flesh. She was quite unable to suggest any cause for her illness, and had sustained no fright or shock of any kind.

On admission on August 18th, 1886, the patient was a thin, slight girl, decidedly anæmic, but with a bright flush on each cheek. She complained of great weakness, and was very excitable, bursting out crying when spoken to. Slight choreiform movements were noticed in the limbs. There was marked exophthalmos, and Von Graefe's lid-symptom was present. The lateral lobes of the thyroid showed great symmetrical enlargement, the vessels over the swelling were dilated, there was visible pulsation in the jugular veins, a thrill could be felt over the tumour, and a loud venous hum was heard.

The neck measured 14½ inches at the greatest circumference. On examination of the chest, the breath-sounds were harsh. There was some pulsation to the right of the sternum and over the cardiac area. The apex-beat was in the left fourth space, with a forcible impulse. First sound at apex short and weak, second at base loud and full; a blowing murmur was heard at the base, loudest over the pulmonary artery, and faintly audible at the apex. The liver-dulness was normal, and nothing was detected in the abdomen. The teeth were carious. Pulse 120, weak, frequent, fairly regular, of low tension. Temperature normal. Urine acid, 1020, no albumen or sugar.

She was ordered ♏ v. of liq. arsenicalis ter die, and a few days afterwards this was increased to ♏ x.

Until September 1st the patient appeared to improve; she slept well, took her food well, and expressed herself as feeling comfortable. But after that date she became rapidly worse, was very sick in the mornings, lost flesh very fast, and was too weak to stand. The arsenic was discontinued on the first appearance of the bad symptoms, and the treatment during the rest of her illness was merely palliative.

Omitting details, on September 10th it was noted that the emaciation had become extreme; there was diarrhœa, but the sickness had stopped; there were sordes on the lips and teeth, bleeding of the gums, and the hair began to fall out rapidly. Pulse 176, small, weak; respiration 36; temperature normal. She still slept well.

Uncontrollable vomiting set in again on the 13th, and at noon of the 14th she lay in a semi-comatose state; there was violent pulsation in the neck and over the heart, and the pulse could not be counted, from its extreme rapidity. Respiration 60. She died a few hours later.

*Autopsy, fourteen hours after death.*—Rigor mortis absent; emaciation extreme; the exophthalmos had disappeared. There was great enlargement of the isthmus and lateral lobes of the thyroid, which latter extended from the root of the neck on each side upwards to some way under the ramus of the jaw; its tissue was firm, but very vascular. The trachea was compressed laterally between the two hypertrophied lobes of the thyroid; its transverse diameter was one-third of an inch, but its calibre was not diminished.

The neck measured 11½ inches at the greatest circumference. There was a small quantity of fluid in the right pleura, and some recent adhesions over the surface of the right lung; the bases of the lungs were œdematous. Pericardium normal. The heart was small, and there was, relatively to its size, some hypertrophy of left ventricle. The valves, endocardium, and substance of walls, were healthy. The thymus was large and persistent, but had the normal appearance; it measured four inches in length and half an inch in thickness. Liver healthy, but weighed only 28 ounces. Pancreas healthy, but extremely small, being four inches long by $1\frac{1}{2}$ inch broad and half an inch thick. Spleen, kidneys, brain, appeared healthy; the latter weighed 45 ounces.

Sections from the pons Varolii, medulla oblongata, highest part of cervical cord, liver, kidneys, pancreas, and thymus, were examined microscopically, and found healthy. Sections from the cervical and first thoracic ganglia of the sympathetic were quite normal, after careful examination as to the state of ganglion cells, nerve fibres, pigmentation, and amount of connective tissue. Sections from the trunk of the vagus appeared healthy under the microscope.

As to the thyroid, it contained a very large number of vessels, I should say, in excess of the normal; the quantity of connective tissue was less than usual, the striking feature about the gland being the very large amount of the epithelial elements in relation to the connective tissue. The individual cells were very distinct. Besides acini of the regular round or oval form, there were large numbers of irregularly-shaped vesicles—doubtless from compression during rapid growth—many of which were elongated and very large. Evidence of rapid proliferation and decay of cells was everywhere present; and in some places—as a sign of their unstable condition—the cells reverted to the columnar instead of the glandular spheroidal type, showing a tendency to return to their original embryological form. Another point was the small extent of the normal colloid degeneration of vesicles present, which would seem to show that the metabolism of the gland had been profoundly altered.

The foregoing appears to be an unique case in medical literature—at least, I can find no report of a similar one—

from the fact that the disease ran an "acute" course; indeed, the patient appeared to waste away under our eyes from day to day.

The patient and her mother were rigidly questioned as to the onset of the disease, and they both positively stated that the illness began only fourteen days before admission; so that—with the exception of the transient attack of palpitation, six weeks previously—the whole duration of the illness was one day less than six weeks.

The other points of interest are, chiefly: the large persistent thymus, two instances of which in patients suffering from exophthalmic goître are recorded by Hilton Fagge; the small size of the liver and pancreas; the very large thyroid; the disappearance of the exophthalmos after death, which perhaps favours the hypothesis that it is due to dilatation of vessels in the orbit, and at any rate is opposed to the theory that it depends on masses of fat in this situation; and the absence of any lesion in the nervous system.

According to our present knowledge, the symptoms included under the term Graves's disease, or exophthalmic goître, vary widely in different cases. The cardinal features of the disease are, palpitation, pulsating bronchocele, exophthalmos, anæmia, and emaciation: all observers agree in stating that in the vast majority of cases palpitation is the first symptom to appear. The ordinary course of the disease is, briefly, the appearance of palpitation, often after a fright, followed at an interval by exophthalmos, and then by a pulsating enlargement of the thyroid, with a peculiar anæmia not relieved by iron, and more or less emaciation. These symptoms, which often seem to be affected by the menstrual periods, generally persist for some years, and the patient probably either recovers, though there is no direct evidence of complete recovery, or more often falls a victim to cardiac failure, to some intercurrent disorder, generally of the lungs, or rarely to suffocation from pressure on the trachea.

*As an instance of recovery to a considerable degree from the disease, I may mention the case of a lady in whom the disease came on in 1871, after a fright experienced during the siege of Paris. All the symptoms mentioned above were present at the outset. The three first were especially intense, and they persisted in their entirety until 1879, when she gradually began to lose both the exophthalmos and the goître, and gained flesh. At the present time she suffers from severe palpitation, anæmia, and from a certain amount of emaciation. She also, after any sudden or too great exertion, is liable to attacks of aggravated palpitation with cardiac syncope; and during these attacks, and for a few days after, the exophthalmos returns, and the thyroid enlarges and pulsates—neither to any very great extent, but sufficiently to become prominent symptoms. So that this case shows that the exophthalmos and the goître may disappear after a time, leaving palpitation, anæmia, and emaciation, and under certain circumstances may reappear for short periods.

In other cases, without the general condition of the patient changing for the worse, exophthalmos and pulsating goître may appear and disappear at different times during the course of the disease, being often intensified at the menstrual periods; and in still other patients either of these two symptoms may be absent, one only appearing in conjunction with anæmia, palpitation, and emaciation.

* NOTE.—Since this patient was under my father's care from 1873 onwards, I have been able to obtain accurate accounts of her illness.

I have also under observation some cases which correspond exactly to Graves's disease in having the characteristic palpitation, anæmia, and emaciation, with the minor symptoms generally complained of, but in whom neither exophthalmos nor goître has ever been present, or only in a very slight and transient degree in the earliest stages of the disease.

These considerations would seem to indicate that exophthalmos and pulsating goître, the prominent clinical features in most cases of Graves's disease, are the least constant if we take the whole number of cases, and that the patient may lose them while retaining the other three chief symptoms; and if in some patients either the exophthalmos or the goître may be absent, and in some both may disappear, it is certainly open to us to argue that in others they may be absent from the beginning.

Again: the frequent absence of one or other of these symptoms, or, as mentioned above, their occasional appearance and disappearance during the course of the disease in varying conditions of the patient, would tend to show that they were not due to a coarse pathological lesion; that even if a lesion were present that satisfactorily explained these two symptoms, it could not be brought forward as an explanation of the more constant features of the disease; and that exophthalmos and pulsating goître presumably depend on a so-called neurosis, or rather on some disturbance in the molecular processes of assimilation and nutrition. Also, since the palpitation occurs in some patients without goître or exophthalmos, it obviously arises either from a different source than the latter arise from, or from one cause acting through different mechanisms; so that, on the hypothesis of a coarse lesion being present, we should require two distinct lesions at least to produce these three symptoms.

Now, the attempts to assign a cause to Graves's disease have been founded for the most part on the assumption that if the exophthalmos, goître, and palpitation were accounted for, the other symptoms would follow; and since in a few cases lesions of the ganglia of the cervical sympathetic have been found, these have been considered by many to be the cause of the disease. The palpitation has been explained by the fact that it may be produced by irritation of the accelerator nerves to the heart, which pass through the third cervical ganglia; and since the vaso-motor fibres from the cervical cord to the thyroid and eyeball also pass through it, destruction of the third ganglia has been thought to produce palpitation by irritation of the first set of fibres, and exophthalmos and goître by paralysis (vaso-constrictor) of the second, causing dilatation of vessels.

Against this may be urged the difficulty of conceiving that one and the same lesion could cause paralysis of one set of fibres and irritation of another set, whilst an irritation lasting for such long periods is unknown in pathology, independently of the fact that the other recognised symptoms of paralysis of the cervical sympathetic are absent.

The lesions described are not altogether of a very satisfactory nature, and it has been objected to them that they might readily have been produced during the processes of preparation for section cutting. They are, increase of connective tissue, changes in the ganglion cells —chiefly pigmentation, opacity of their protoplasm, and shrinking of the cells from their capsules. In a large number of sympathetics that I examined the amounts of pigmentation and connective tissue varied greatly, and the ganglion cells did not always accurately fill their capsules, and appeared somewhat opaque, in cases where there was no reason to suspect sympathetic disease.

A case has been recently reported by Hack* where exophthalmic goître seemed to depend on a neurosis of the sympathetic, from irritation of its peripheral filaments in the nose; there was extreme hypertrophy of the erectile tissue over the middle and lower turbinated bones. This was removed by operation; and several months after, the exophthalmos and palpitation had disappeared, and the goître diminished in size. This case, so far as it goes, is perhaps in favour of the cervical sympathetic being the seat of origin of the exophthalmos, goître, and palpitation; though the recovery does not seem to have been complete, and enough time has not elapsed since the operation to speak with confidence as to the future state of the patient. Experiments with a view to produce these symptoms by injury or destruction of the cervical ganglia have, I believe, failed.

In a few cases† diabetes occurred in patients suffering from exophthalmic goître; and since diabetes has been produced by destruction of the first thoracic and third cervical ganglia, this has been brought forward as an argument in favour of a lesion in this region of the sympathetic. The experiments by which diabetes was produced by injury or removal of these ganglia were, however, very contradictory—so much so, that no satisfactory conclusions can be drawn from them.‡

* *Deutsche med. Wochenschrift*, June 24th, 1886.

† Especially one reported by Dr. Lauder Brunton, St. Bartholomew's Hospital *Reports*, vol. x.

‡ Dr. Michael Foster, *Text-book of Physiology*, 3rd ed., p. 391.

Finally, Wilks, Paul, Fournier, and Ollivier, assisted by Ranvier, Ross,* and others, found nothing abnormal in the cervical or other parts of the sympathetic; so that, as Ross remarks, the pathological evidences of sympathetic lesion as the cause of Graves's disease are at least doubtful, and to be established as the *fons et origo mali* must be supported by independent evidence.

Such independent evidence, however, has seemed to some authors—Brown-Séquard, Benedikt, Long Fox, and others—to indicate that the cause of the disease must be sought for in the medulla oblongata and upper cervical cord; and I shall try to show that this is the most satisfactory hypothesis. Now, another way in which diabetes may be produced is by the well-known diabetic puncture in the medulla, at a spot corresponding pretty closely to the great vaso-motor centre in this region; producing diabetes, probably, by dilatation of the hepatic arteries. Close to this spot is another important centre, the cardio-inhibitory, whose function it is, through the pneumogastric, to exercise a constant regulating and slowing influence on the heart-beats; and if this centre ceases to act properly, the heart at once begins to beat rapidly in palpitation. It seems to me that palpitation in Graves's disease is better explained by deficient action of this centre than by any other cause.

Again: the marked loss of vaso-motor tone, showing itself by the general relaxation of arterioles that is constantly present in the disease, could be accounted for at first by some depressing influence acting on the adjacent vaso-motor centre; and the occurrence of diabetes in these few cases seems to throw light on exophthalmic goître by showing that the medullary centres, rather than

* Ross, *Diseases of Nervous System*, vol. i., p. 715.

the cervical sympathetic, are the parts at fault. Also, it was mentioned by Dr. Long Fox, at the discussion following this paper, that exophthalmos, pulsating goître, and palpitation have been produced by section of the restiform tracts.

Taking the symptoms of Graves's disease, then, in the order of their onset in the great majority of cases: The first evident symptom is palpitation, which may be explained, as above, by a deficient action of the cardio-inhibitory centre, produced in some way, most probably by antecedent changes in the molecular nutrition of the cells quite sufficient to produce serious disturbance of function, without leaving results appreciable by our present means of investigation. And this palpitation may be but the first outward and visible sign of some profound changes of metabolism and assimilation already established in the patient. Possibly this primary disturbance in the nutritive processes of the organism may be induced by changes in the metabolism of the thyroid—for we have strong evidences of change in this gland—which may either pour into the blood some product of mal-assimilation injurious to the sensitive cells of the medullary centres, or allow to accumulate in it some body which normally it should remove.

However this may be, we may surmise that the first change is some abnormality of the processes of assimilation and nutrition in the patient; and in the case I have read there is striking evidence of this in the remarkably small size of the liver and pancreas, and large bulk of the altered thyroid and persistent thymus. Paresis, or depression of the cardio-inhibitory and vaso-motor centres following on this mal-nutrition, produces palpitation and relaxation of arterioles respectively in the earliest stages of the disease; while the exophthalmos and pulsating goître may be caused by a similar process extending to subordinate vaso-motor centres in the cervical spinal cord for the eyeball and thyroid,—exophthalmos being also probably caused in part by stimulation of the muscle of Müller.

After the earliest stages of Graves's disease, there is in most cases considerable anæmia; and since in anæmic persons palpitation occurs from relaxation of the arterioles, due to loss of vaso-motor tone, giving rise to disproportion between the action of the heart and the work it has to do, this anæmia would increase the palpitation and loss of arterial tone already produced; whilst, acting in a vicious circle, the two latter would again favour increased anæmia.

To this hypothesis of the rationale of the disease the fact that the other functions of the cervical sympathetic are normally performed is in no way opposed, while it is a formidable objection to the theory that attributes the prime cause of the disease to a lesion of the nerve in this situation. We can also explain the cases in which either exophthalmos or pulsating goître is absent by saying that in these patients the main centres in the medulla are alone involved, and for some reason the subordinate centres generally implicated in exophthalmic goître have escaped, the disturbance not extending to them; and since only a functional derangement, a disturbance of molecular nutrition in the cells, is postulated, we can understand that (even in cases in which the symptoms were originally severe) partial, as in the patient mentioned above, or perhaps complete recovery may take place—facts which would certainly be difficult of comprehension if the disease depended on a definite destroying lesion in any part of the nervous system. Moreover, the undoubtedly beneficial effects of arsenic in Graves's disease receive explanation.

In conclusion, returning for a moment to the case which forms the subject of this paper, in this girl, who died after an attack of such unusually short duration, there seems to have been an intensification, or, if I may so call it, a "malignant" type of Graves's disease; for the rapid and unaccountable wasting that took place was quite out of proportion to the vomiting and diarrhœa that occurred, and to be explained only by some profound disturbance of the processes of nutrition, to which the *post-mortem* appearances also point.

